# Fungal infection has sublethal effects in a lowland subtropical amphibian population

**DOI:** 10.1186/s12898-018-0189-5

**Published:** 2018-09-14

**Authors:** Laura A. Brannelly, Matthew W. H. Chatfield, Julia Sonn, Matthew Robak, Corinne L. Richards-Zawacki

**Affiliations:** 1 Department of Biological Sciences, University of Pittsburgh, Pittsburgh, PA USA; 2 School of Biodiversity Conservation, Unity College, Unity, ME USA; 3 Department of Ecology and Evolutionary Biology, Tulane University, New Orleans, LA USA

**Keywords:** Capture-mark-recapture, Chytridiomycosis, Disease tolerance, Wildlife disease

## Abstract

**Background:**

The amphibian chytrid fungus, *Batrachochytrium dendrobatidis* (*Bd*), has been implicated as a primary cause of decline in many species around the globe. However, there are some species and populations that are known to become infected in the wild, yet declines have not been observed. Here we conducted a yearlong capture-mark-recapture study and a 2-year long disease monitoring study of northern cricket frogs, *Acris crepitans*, in the lowland subtropical forests of Louisiana.

**Results:**

We found little evidence for an impact of *Bd* infection on survival; however, *Bd* infection did appear to cause sublethal effects, including increased capture probability in the field.

**Conclusions:**

Our study suggests that even in apparently stable populations, where *Bd* does not appear to cause mortality, there may be sublethal effects of infection that can impact a host population’s dynamics and structure. Understanding and documenting such sublethal effects of infection on wild, seemingly stable populations is important, particularly for predicting future population declines.

**Electronic supplementary material:**

The online version of this article (10.1186/s12898-018-0189-5) contains supplementary material, which is available to authorized users.

## Background

Emerging infectious diseases have become an important factor in understanding wildlife health over the last few decades [1]. Some of the most impactful infectious diseases of wildlife have been fungal, and these have caused catastrophic declines and even extinctions in both plants and animals [2]. The fungal disease chytridiomycosis, caused by the fungal pathogen *Batrachochytrium dendrobatidis* (*Bd*), affects amphibians specifically and has been named one of the most devastating wildlife diseases in recorded history [3]. Chytridiomycosis is known to have caused population declines in a number of susceptible frog species around the world [4, 5]. However, less is known about its impacts on less-susceptible species, i.e., those that are infected in the wild but show no evidence of decline due to disease.

Capture-mark-recapture (CMR) studies can be a powerful tool for clarifying the impact of *Bd* infection on wild populations [6–11]. CMR studies permit the estimation of survival, capture and infection state change probabilities, and population size through animal capture data and the inclusion of a variety of biotic or abiotic variables [12]. CMR studies on *Bd*-susceptible species have been used to demonstrate that even when chytridiomycosis has reached an enzootic state within a population, survival can be substantially reduced in *Bd*-infected animals compared to uninfected animals [6–11]. While understanding disease dynamics within susceptible and declining populations is important for conservation purposes [10, 13], investigating disease impacts on apparently stable populations is also important for understanding the potential for less dramatic yet still meaningful impacts of this pathogen on host ecology. CMR analyses can be used to estimate whether a population is stable, crashing or rebounding. Furthermore, these analyses can be used to predict which circumstances might lead to disease outbreaks or disease-induced population crashes [14, 15]. To date, limited research has investigated the effects of *Bd* infection on apparently stable host populations.

*Acris crepitans*, the northern cricket frog, is native to the Southeast, Midwest and Northeast regions of the United States. The species is listed as least concern by the IUCN [16], and throughout much of the species’ range it is abundant. There have been large scale declines reported, particularly in the subspecies *A. c. blanchardi*, which were first noticed in 1977 in southern Ontario, Canada, and have since been documented across much of its northern distribution [17]. These declines have been hypothesized to result from habitat modification and loss, habitat acidification, and toxins [18, 19]. *Bd* is known to infect *A. crepitans* in the wild [20–22], and mortality due to chytridiomycosis has been documented ([20]; authors’ pers. obs.), but chytridiomycosis has not been directly implicated in the declines that have occurred.

In this study we used field surveys to assess the impact of *Bd* infection on *A. crepitans* from the southern extent of its range in Louisiana. The lowland subtropical population we studied is known to harbor *Bd*, but animals remain abundant. We expect impacts of *Bd* to be minimal in this population due to warm summer temperatures in the region, which are outside the thermal optimum for *Bd* growth. Further, *A. crepitans* is a diurnal species [23], which may increase its basking behavior compared to other species in the region thereby reducing infection. We conducted a CMR study of a single population of *A. crepitans* for 1 year in order to assess the impact of *Bd* on the population. We then continued to monitor for an additional year, sampling for infection prevalence and intensity, as well as comparing *Bd* dynamics in this host with that in other amphibian species found at the site. We aimed to determine the impact of *Bd* on survival and capture probability across seasons, as well as compare yearly infection dynamics of *A. crepitans* with other sympatric species to better understand the impacts of *Bd* infection on wild populations.

## Methods

### Study site

**Fig. 1:**
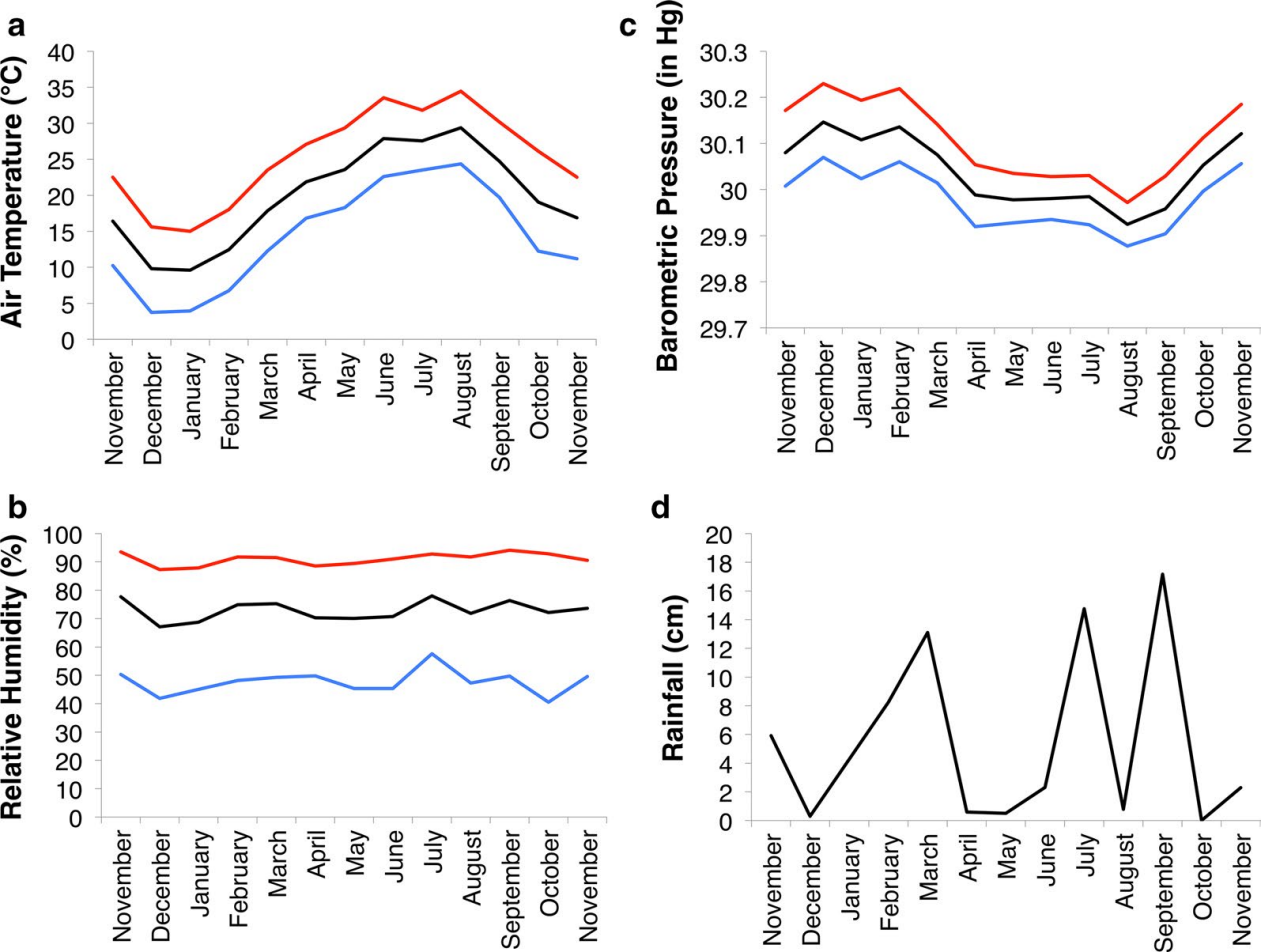
Monthly averages of weather variables. Air temperature (**a**), relative humidity (**b**), barometric pressure (**c**), and cumulative monthly rainfall (**d**) as measured by the nearest weather station (9.9 km SW of field site), from November 2010–November 2011. Daily mean is in black, maximum is in red, and minimum is in blue

We conducted our field study at Tulane University’s F. Edward Herbert Research Center in Belle Chasse, Louisiana (29°52′29.4″ N, 89°55′16.2″ W, elevation 2 m). The study site was an approximately one acre wooded ephemeral pond 200 m from a bayou. *Acris crepitans* at this site are active and breeding year-round [24], whereas other species use the site only when the pond contains water. During each survey the entire pond depression and surrounding wooded area was searched visually and haphazardly for all amphibian species. At least two people searched the site for at least three full hours on each survey day. We sampled frogs at the site twice per month, on 2 non-consecutive days, during daylight. Data from a nearby weather station (Naval Air Station Joint Reserve Base New Orleans, located 9.9 km SW of study site) for the corresponding time periods was obtained from Weather Underground, Inc. (The Weather Company, IBM) (Fig. 1).

### Seasonal infection prevalence and intensity

From November 2010 through July 2012, we sampled all post-metamorphic amphibians encountered during our surveys to estimate *Bd* infection intensity and prevalence. We captured each animal with a clean, nitrile-gloved hand and placed it in a new, individual plastic zip bag. Shortly thereafter (within 1 h), we swabbed the animal for *Bd* (described below). We placed animals in a cool space away from direct sunlight and released them where they were captured after the survey was complete to avoid capturing an animal twice on the same survey day.

### Capture-mark-recapture study

From November 2010 to November 2011 we sampled *A. crepitans* within the designated study site for capture-mark-recapture analysis. After data was collected, as described above, we used a toe tip removal scheme to individually identify each animal [25, 26]. Toe tips were removed with disinfected surgical blades, where no more than one toe per foot and four toes total were removed per animal. The thumb was never removed. While there has been some recent concern over the use of toe-clipping as a marking method for amphibians, there is no evidence that the marking scheme used in this study causes any more harm than that of temporary handling stress [27–29], and toe-clipping can be the safest and most reliable marking scheme for small amphibian species [26].

While surveys were conducted twice a month, each month’s captures were combined together to minimize dispersion of the data. If an individual was captured twice in 1 month, the infection load (measured in zoospore equivalents, or ZE) was averaged (n = 6) between the two samples. If the animal was negative for *Bd* (n = 25), the infection load was equal to 0 ZE, therefore if an animal was captured twice and one capture was a positive and the other was a negative, the samples were still averaged (n = 10).

### Testing for *Bd*

To test for *Bd*, we swabbed (with swab model MW113, Medical Wire and Equipment Co.) each animal’s skin five times on each of the dorsum, venter, sides, and undersides of each foot. Swabs were stored dry at − 20 °C prior to DNA extraction. We extracted genomic DNA from swab samples using the DNeasy Blood and Tissue Kit (Qiagen, Inc.) following the protocol for animal tissue, but with the following modifications: swabs were incubated for 1 h and were vortexed and spun in a centrifuge once after 30 min and again at the end of the incubation period, and samples were eluted twice using 100 µL of elution buffer each time for a final elution volume of 200 µL. We then used a quantitative polymerase chain reaction (qPCR, Applied Biosystems) assay to detect and quantify *Bd* DNA in these extracts. Our assay followed Boyle et al. [30] with the following modifications: we did not dilute the DNA extracts and included both an internal positive control [31] and bovine serum albumin (BSA) [32] in each reaction well. Each run contained positive and negative controls (extracted from swabs of known *Bd*-positive and *Bd*-negative captive *Rana catesbeiana*) and a five-fold dilution series of standards made using a solution of zoospores of the JEL423 *Bd* isolate. We tested each extract only once (singlicate) to maximize cost effectiveness [6, 33], and considered a sample positive for *Bd* if the qPCR indicated > 1 ZE in the reaction. ZE was calculated as the number of zoospores present in the whole extract (whole swab value).

### Analysis

#### Infection dynamics

To test for differences in infection prevalence (the proportion of infected individuals) and intensity (the pathogen load, or ZE calculated from the qPCR results) in *A. crepitans* over time, we used qPCR results from the full 22 months of surveys (10 months beyond the end of the CMR study). To test for variation in infection prevalence across months and years we used a generalized linear mixed model (GLMM) using a binary logistic regression. Infection status (*Bd*-negative/positive) was the dependent variable, and month, year and the interaction between month and year were fixed effects. Individual ID was included as a random effect (Additional file 1).

To test for differences in infection intensity of *Bd*-positive *A. crepitans* over time we used a mixed effects model, and we used only *Bd*-positive captures in this analysis. Zoospore equivalents were log_10_ transformed, and this was the dependent variable. Month and year of capture and their interaction were included as fixed effects in the model, and individual ID was a random effect.

We also tested for differences in pathogen load among species over the same survey period. Some species had few positive animals so infection intensity and prevalence were combined to assess pathogen load. Pathogen load was analyzed among species including both the positive and negative animals of each species. We conducted a GLM where infection intensity (log_10_ (ZE + 1)) was the dependent variable, and fixed factors were year, month and species. A second test was conducted on all species except *A. crepitans* with the same variables. This second test was conducted because there were many more *A. crepitans* collected over the study period, and we wanted to make sure the patterns observed when all species were included were not unduly influenced by the inclusion of this better-sampled species. Species were only included in both of these analyses if at least 10 individuals were captured over the study period (Additional file 2). All of the above analyses were performed in SPSS v21.

#### Population modeling

We estimated survival (Φ), capture (p), and disease state transition (Ψ) probabilities for *A. crepitans* adult animals using our CMR data of post-metamorphic capture and swab data from the frogs during the 12-month survey period using the Conditional Arnason–Schwarz model in M-SURGE [34]. M-SURGE is a program designed specifically for multi-state CMR studies, and its outputs have been shown to be robust even with low recapture rates [34]. The variables included in our model set were time in months (t), *Bd* state at capture (f), and *Bd* state at previous capture (to). The two disease states were *Bd*-positive and *Bd*-negative, based on the swab data collected with each capture. In this model, the types of disease state transitions that are predicted are: (1) remain *Bd*-negative, (2) become infected (transition from *Bd*-negative to *Bd*-positive), (3) remain *Bd*-positive, and (4) clear infection (transition from *Bd*-positive to *Bd*-negative). All variables and combinations of variables (additive and multiplicative) were assessed for each term probability in order to generate the best-fit model. We measured goodness of fit (ĉ) with the program U-CARE [35] (Additional file 3).

We estimated the population size for *A. crepitans* using a POPAN model [36] in Program MARK [37]. The POPAN model is a formulation of the Jolly-Seber model for CMR analysis that estimates survival and capture rates as well as abundance and recruitment rates. The POPAN model additionally estimates a “super-population” size (the theoretical finite populations size estimated via population modeling number—the subpopulation size of the animals exposed to sampling, which represents an estimate of the total population size at a site). The specific probability estimates in the model set were survival probability (Φ), capture probability (p), and probability of entry into the population (P_ent_). These estimates are derived from the individual capture data only; they do not account for disease status. Variables that were considered to affect these probabilities were time in months (t), or no variable (.). Population (or super-population) size was estimated using the best-fit model in Program MARK. All possible model variables were analyzed separately in order to determine the best-fit model. Data dispersion was assessed using U-CARE, as stated above.

We used Akaike’s information criterion (AIC) for model selection, where the best fitting model is indicated by the lowest AIC value, or within two units from the lowest AIC value (ΔAIC < 2.0). We selected the eight best-fitting models as well as the full (all variables included) model and the null model, and all the models tested that converged for the POPAN model (Table 2).

Model averaging was performed if more than one model was within 2 AIC values of the best fitting model. Model averaging was performed by calculating model weights [38, 39] (reported in Table 2), then using the outputs of the best fit models for survival, capture and state change estimates, average model outputs were calculated following the “natural averaging” approach [38].

## Results

### Infection dynamics in *A. crepitans*

**Fig. 2:**
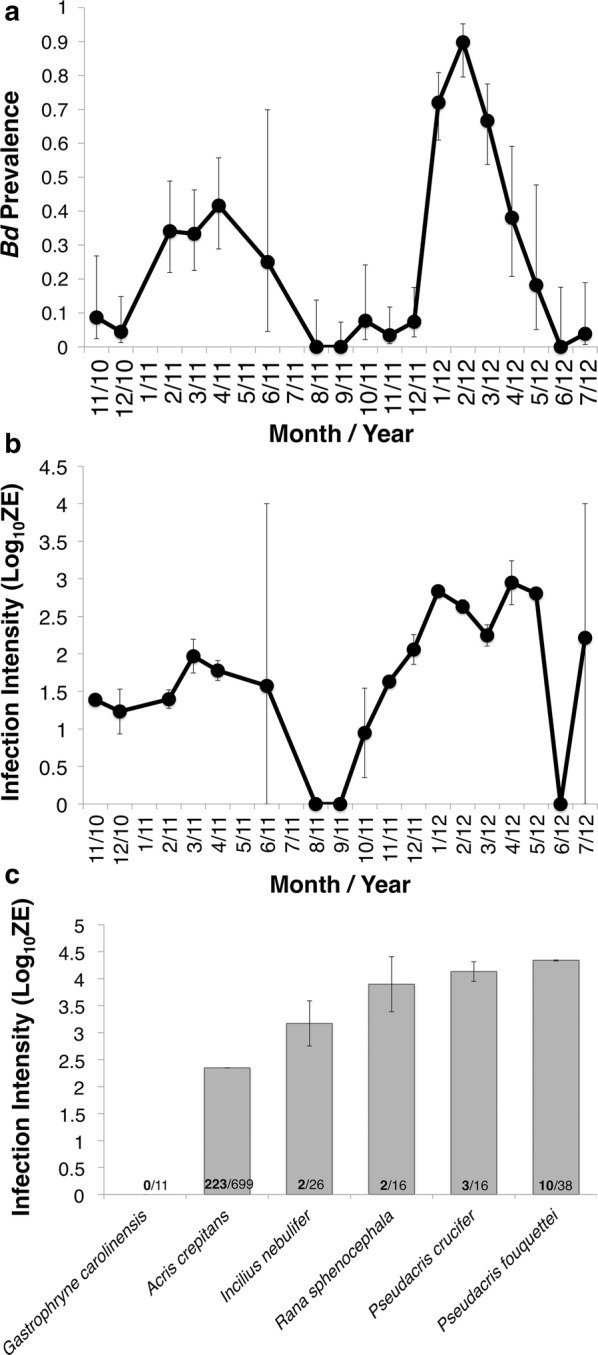
Infection prevalence and intensity over the field survey period, November 2010–July 2012. **a** Proportion of *A. crepitans* infected with *Bd* is on the primary y-axis (black), and number of individuals captured each month is on the secondary y-axis (grey). Error bars indicate 95% confidence intervals. **b** Mean infection intensity of *A. crepitans* across months, measured as log transformed zoospore equivalents of only infected individuals and the error bars indicate standard error. **c** Infection intensity of all species collected, averaged and including the negative samples. Error bars indicate standard error. Numbers at the base of the bars represent the number of animals that tested positive over the total number of animals sampled

Over 22 months of field surveys (November 2010 to July 2012), we collected skin swabs for *Bd* analysis from 699 capture events of *A. crepitans.* Infection prevalence varied across months (GLMM: Month, F_11,681_ = 7.63, p < 0.01), and there was also a significant interaction between month and year (GLMM: month * year, F_4,681_ = 5.067, p < 0.01), but year alone was not a predictor of infection prevalence (GLMM: year, F_2,681_ = 0.351, p = 0.704). Prevalence was highest between January and May and lowest between July and November. Infection prevalence was higher January through March of 2012 than for those same months in 2011 (Fig. 2a).

Infection intensity varied across years (mixed effects: year, F_2,206.72_ = 21.721, p < 0.01) but not months (mixed effects: month, F_9,177.53_ = 1.485 p = 0.16), though the interaction between month and year was also significant (mixed effects: month * year, F_3,202.12_ = 4.379, p < 0.01). The 2nd year of the survey had a higher mean infection intensity than the first, and this pattern appears to have been driven by an increase in infection intensity in the cooler months (January–May; Fig. 2b).

#### Infection dynamics in other amphibian hosts

In addition to the 699 *A. crepitans* captures described above, we captured 38 *Pseudacris fouquettei* (Cajun chorus frog), 26 *Incilius nebulifer* (gulf coast toad), 16 *Pseudacris crucifer* (spring peeper), 16 *Rana sphenocephala* (southern leopard frog), and 11 *Gastrophryne carolinensis* (eastern narrow-mouthed toad) over the course of our study. When all species were included in the analysis, pathogen load differed across months and species (GLM: month, F_11,794_ = 12.501, p < 0.01; species, F_5,794_ = 5.413, p < 0.01) and the interaction between month and species, as well as between month and year were significant (GLM: species * month, F_15,794_ = 5.009, p < 0.01; month * year, F_5,794_ = 7.749, p < 0.01), but year alone and the interaction between year and species and all three factors did not affect pathogen load (GLM: year, F_2,794_ = 0.284, p = 0.75; year * species, F_4,794_ = 0.226, p = 0.92; year * species * month, F_2, 94_ = 0.73, p = 0.482). When *A. crepitans* was not included in the analysis, pathogen load differed across months and the interaction between month and species was significant (GLM: month, F_7,95_ = 6.196, p < 0.01; month * species, F_8,95_ = 2.683, p = 0.013), but main effects of year, species and all other interactions were not significant (GLM: F_< 4,95_ < 0.54, p > 0.75 in all cases). *Acris crepitans* had lower infection intensity than some other species, such as *P. fouquettei*. Although our sample size was small, we did not detect *Bd* on any *G. carolinensis* (Fig. 2c).

#### Multistate capture-mark-recapture

**Fig. 3:**
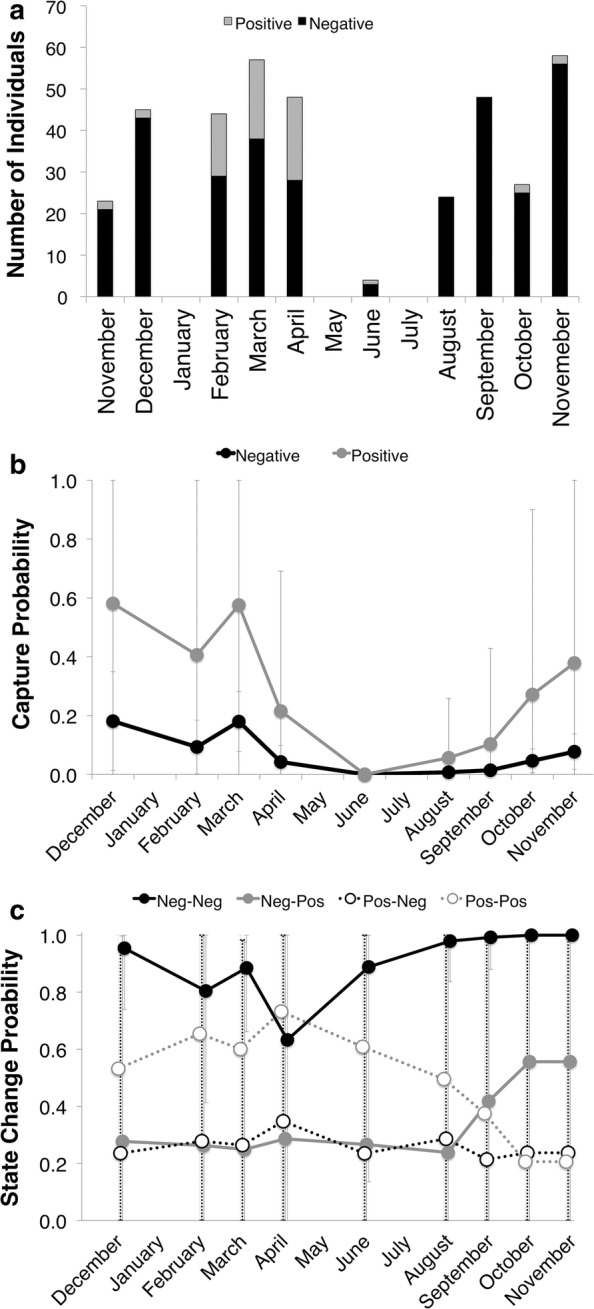
Capture-mark-recapture results over from November 2010–November 2011. Panel **a** shows the proportion and number of *Bd*-positive and -negative *A. crepitans* captured each month of the capture-mark-recapture study. **b** Shows monthly capture probabilities after model averaging, where the variables are time and disease state. **c** Shows *Bd*-state change probability, where month, *Bd*-state of previous capture, and *Bd*-state of capture are included. State change probability is the probability that an individual will change state before the next capture. Four state changes are possible: staying negative (neg–neg), gaining infection (neg–pos), clearing infection (pos–neg), and remaining infected (pos–pos). All error bars indicate 95% confidence intervals

We captured 266 individual *A. crepitans* at least once during the CMR portion of the study (Table 1). Fifty of these individuals were recaptured at least one time, and 13 were recaptured twice for a total of 378 capture events between November 2011 and November 2012 (Table 1). Sixty-three capture events were of *Bd* positive animals (Fig. 3a). Analysis of dispersion of the data (goodness of fit) yielded ĉ = 0.788, and as such ĉ was not adjusted in the model.

**Table 1:** Summary of the *A. crepitans* capture-mark-recapture data

CMR data	Instances
Data summary	
Unique individuals captured	266
Number of animals recaptured	50
Positive captures	315^a^
Negative captures	63^a^
Disease state change of recapture^a^	
Stay negative	41
Negative to positive	12
Positive to negative	6
Stay positive	4
Negative to positive to negative	1

^a^Total number of capture instances, where some individuals are represented more than once

The three best fitting Conditional Arnason–Schwartz models (Table 2) for this study were averaged. The monthly survival estimate (Φ) was 93.13% (95% CI 82.64–100.00%) for uninfected animals and 92.23% (95% CI 64.47–100.00%) for infected animals.

**Table 2:** Best fitting parameterizations of the Conditional Arnason–Schwarz and POPAN Models

Model	# of parameters	Deviance^1^	AIC^2^	ΔAIC^3^	AIC weights^4^
Conditional Arnason–Schwarz model^a^					
Φ(.)p(to+t)Ψ(f * to + t)	21	487.1455	*529.1455*	0	0.4210
Φ(.)p(t)Ψ(f * to + t)	20	490.8493	*530.8493*	1.7038	0.1796
Φ(f)p(to + t)Ψ(f * to + t)	22	487.1309	*531.1309*	1.9854	0.1560
Φ(f)p(t)Ψ(f * to + t)	21	489.683	531.683	2.5375	0.1183
Φ(t)p(t)Ψ(f * to + t)	28	480.4241	532.4241	3.2786	0.0817
Φ(.)p(to + t)Ψ(t)	20	495.8178	535.8178	6.6723	0.0150
Φ(t)p(to + t)Ψ(f * to + t)	29	477.9171	535.9171	6.7716	0.0143
Φ(.)p(to + t)Ψ(i)	12	511.9563	535.9563	6.8108	0.0140
Φ(f * t)p(to * t)Ψ(f * to * t)	54	460.0165	546.0165	16.871	0
Φ(.)p(.)Ψ(.)	3	581.9903	587.9903	58.8448	0
POPAN model^b^					
Φ(.)p(t)p_ent_(t)	14	− 1028.251	*417.6716*	0	0.9962
Φ(t)p(t)p_ent_(t)	20	− 1030.5198	428.8351	11.1635	0.0038
Φ(t)p(.)p_ent_(t)	15	− 1004.0417	444.0828	26.4112	0
Φ(.)p(.)p_ent_(t)	9	− 962.111	473.0152	55.3436	0
Φ(.)p(.)p_ent_(.)	3	30,658.918	32,081.5399	31,663.8683	0
Φ(t)p(t)p_ent_(.)	15	30,659.738	32,107.8919	31,690.1903	0

Italicized terms are AIC values indicating the best fit models

^1^Deviance is a measure for how well the model fits the data

^2^Akaike’s information criterion

^3^Change in AIC from the single best fit model

^4^AIC weight is the model probability within the candidate model set

^a^Probabilities estimated are survival (Φ), capture (p), and disease state change (Ψ); and the variables examined to influence Φ, p and Ψ were time in months (t), *Bd* state at previous capture (to), *Bd* state of capture (f) and no variable (.)

^b^Probabilities estimated are Φ, p, and probability of entry into the population (P_ent_); these were modeled as either dependent upon time (t) or no variable (.)

Month of capture (t) influenced capture probabilities (p), which were lower in the warm months (May, June and July) than in the cool months (December, January and February) of the year (Fig. 3b). The animal’s *Bd* state of previous capture (to) affected capture probability as well, where infected animals were more likely to be captured (Fig. 3b).

*Bd* state change probability varied across months (t) and with the animal’s *Bd* state at previous (to) and current (f) capture in all three of the best fit models (Table 2; Fig. 3c). *Bd*-negative animals had a very high likelihood of remaining *Bd* negative, and *Bd*-positive animals had a higher probability of remaining *Bd*-positive in the early months of the year (December–June), and a lower probability of remaining *Bd* positive in the fall (September–November). Animals that were infected had a consistent, low likelihood of clearing infection throughout the study, and animals that were *Bd*-negative were more likely to become infected in the fall months (September–November).

#### Population size

The best fitting POPAN model was Φ(.)p(t)p_ent_(t) (Table 2). Monthly survival (Φ) was not influenced by month (t), while both capture probability (p) and monthly entry into the population (p_ent_) were influenced by month (t). The population size estimate, based on the best fitting model for the survey period, was 1854.33 (95% CI 1092.28–3323.45). Dispersion analysis of the data showed near perfect dispersion: ĉ = 0.979, and ĉ was not adjusted in the model.

## Discussion

In this study our aim was to determine the impact of *Bd* on a lowland subtropical population of *A. crepitans*; a population known to harbor *Bd*, but where animals remain abundant. We assessed the survival and capture probability of *A. crepitans* across seasons, as well as compared yearly infection dynamics with other sympatric species to better understand the impacts of *Bd* infection in wild populations. Through the infection dynamics field study, we found that seasonality plays a large role in infection dynamics in *A. crepitans*, with higher infection load and intensity in the cooler months (January–May), and near zero infection in the warmer months (July–November). In addition to seasonal infection dynamics in *A. crepitans*, we also found seasonal fluctuation in the other sympatric species, where pathogen prevalence varied greatly across months. These results support the seasonality of *Bd* infection dynamics that other studies have demonstrated, presumably because cooler temperatures are better for the pathogen’s survival and reproduction [7, 21, 40–43].

In this study we found higher intensities of infection in *A. crepitans* in the second year of the survey. Year-to-year fluctuations are also common with this pathogen, which could be due to abiotic factors such as changes in weather patterns (e.g., temperature or rain fall) from year to year. The fluctuations could also be due to biotic factors such as shifts in amphibian population size. In many amphibian species not experiencing declines, population size varies yearly based on recruitment success and other factors [44]. Because *Bd* dynamics have shown density-dependence [6, 8], it follows that *Bd* infection dynamics would then vary year-to-year as well [45].

Our comparisons of infection between the species captured suggest that *A. crepitans* has a lower overall pathogen load than the other species surveyed in this study. This result could stem from the overrepresentation of *A. crepitans* in our dataset (and thus smaller error bars for this species). Our study design does not permit us to assess which species are driving infection dynamics in this community. The mechanisms of pathogen transmission in the amphibian-*Bd* system [46, 47], and in particular what maintains pathogen presence following warm summer months when the animals appear to clear infection, remain unclear. It is possible that there is an environmental reservoir for *Bd*, but the type of long-term environmentally-stable life stage needed to enable this has so far not been detected for this pathogen [48].

A second major aim of this study was to understand the impacts of *Bd* infection on wild animals in the lowland subtropics, where some species are known to harbor *Bd* infection but remain abundant. We used CMR analysis to estimate the effects of *Bd* exposure on survival, capture and disease state change probability in a wild population of *A. crepitans*. For estimates of survival, we found that while disease state was a variable in one of the best fitting models, the monthly survival values were nearly identical for infected and uninfected animals (< 1% difference in estimated mean monthly survival), with wide and overlapping 95% confidence intervals. The small mean difference in estimated survival, suggests that the difference in survival between infected and uninfected animals is not biologically meaningful [49, 50]. What is noteworthy about these survival estimates is they demonstrate that *A. crepitans* has a high monthly survival probability (~ 0.93), which suggests that the annual survival is on the order of ~ 0.419. In more northern populations, *A. crepitans* is known to have low annual survival, with complete population turnover occurring between 16 and 24 months [51, 52]. In the subtropical lowlands amphibians do not undergo periods of prolonged cold temperatures, and perhaps this contributes to higher annual survival rates.

While we found little to no support for an effect of infection status on survival probability, we did find evidence for sublethal effects of infection. Through our CMR study we found that while capture rates vary seasonally, there is also a higher capture probability for infected animals than for *Bd*-negative animals. This result suggests that *Bd* infection may cause the animals to alter their behavior in some way that makes them easier to capture. Other CMR studies investigating *Bd*–host dynamics have similarly found that infected animals were more likely to be captured, and even more so when pathogen load is high [6, 7, 9].

There have been few documented instances of sublethal effects of *Bd* infection on populations and species that are not experiencing mortality due to *Bd*. When individuals that do not succumb to chytridiomycosis are exposed to *Bd*, and either tolerate infection or clear infection, the immune system is activated [53–55]. As the immune system is activated, there should be measurable sublethal effects of infection. While there has been considerable research devoted to amphibian immune function in susceptible and non-susceptible hosts (e.g., [56–59], there are few studies that measure the sublethal effects of *Bd* infection on less susceptible or resistant hosts. Weight loss after *Bd* infection but without mortality has been demonstrated in a newt species, *Lissotriton helveticus*, [60] and the European common frog, *R. temporaria* [61]. There is a decrease in reproductive effort following infection in newts, *L. helveticus* [60], and, in adult leopard frogs, *R. pipiens*, there is a decrease in jumping ability following exposure to *Bd* [62]. These documented physiological changes after exposure to *Bd* may result in behavioral changes such as reduced escape behavior. In tadpoles, exposure to *Bd* has been shown to cause changes in anti-predator behavior [63, 64]. The differences in capture probability observed in this study between infected and uninfected individuals, particularly in the cold months of the year when infection is much higher, may be due to changes in physiology or behavior as a result of infection. Such sublethal effects from stressors such as infection could lead to changes in individual fitness such as reduced growth, foraging ability, and reproduction [65–67], which could ultimately change population dynamics in subtle ways over time.

The seasonal variation in capture probability that we observed, with lower capture rates in the warmer months than in the cooler months, is typical of subtropical amphibians [68]. These shifts in capture probability appear to occur when temperatures begin to warm and barometric pressure is low (Fig. 1). In the subtropical lowlands of Louisiana, summer months are hot and can be dry, particularly during the heat of the day, leading amphibians to aestivate [69], which would decrease capture probabilities. Amphibian activity is often linked with ideal temperatures and rainfall, and in the subtropics, frog activity is often opposite to that of temperate frogs, which are active during the summer and hibernate during the winter.

The state change probabilities we observed match the seasonality of *Bd* infection. There is a higher likelihood of remaining *Bd*-positive between December and June (Fig. 3c) when infection prevalence is high (Fig. 2b, c), and a lower likelihood of maintaining infection in the fall (September–November). After model averaging the 95% confidence intervals for state change probability remained large. This could be due to the few animals recaptured with *Bd* infection (Table 1). However, the error bars are smaller for the probability of remaining *Bd*-negative, and the seasonal pattern supports what is known about the seasonality of *Bd* infection; animals are less likely to be *Bd*-negative in the spring (March–April) when temperatures are cool, and more likely to be *Bd*-negative free in the fall (August–November) after the heat of the summer in subtropical environments [7, 21, 40, 41].

While infection status appears to have impacted capture probability in this *A. crepitans* population, we found no evidence that it impacted survival. The model-averaged estimates for survival in *Bd*-positive and *Bd*-negative animals are very close in value and their confidence intervals show a large degree of overlap. Given this, we find no evidence for an effect of *Bd* infection on survival in this population. Lowland subtropical habitats often have shorter periods of infection [70], which might lead to infected animals clearing infection before they succumb to disease, and thus minimize direct impacts on survival, but still cause sublethal effects of infection.

*Acris crepitans* has declined in parts of its range, particularly in the northern regions. While *Bd* has not been specifically implicated in the declines that occurred, this frog species is known to be susceptible to chytridiomycosis under certain conditions [71]. Our results suggest that in this subtropical population *Bd* is not affecting survival, but may have sublethal effects in the form of changes to behavior of infected animals.

## Conclusions

Our study aimed to determine the impact of fungal infection on a frog population where disease is endemic, but animals apparently remain abundant. Through our capture-mark-recapture analysis we found that in a lowland subtropical population of *A. crepitans* there was little effect of *Bd* on survival. However, there was a clear difference in capture probability, which may indicate a shift in behavior as a result of infection. Evidence for sublethal effects of *Bd* infection is rare in wild populations not experiencing mortality, and finding it here suggests that *Bd* may be impacting *A. crepitans* in populations that remain abundant and where no reported declines are occurring. Because amphibians are declining globally, monitoring seemingly stable populations is important, especially for a species that has declined elsewhere. Our work demonstrates that while survivorship may not be impacted by disease, behavior can be, which may lead to subtle shifts in population dynamics over time.

## Additional files

**Additional file 1.** Capture details for each *Acris crepitans* captured in the study.

**Additional file 2.** The swab results of all species except *Acris crepitans*.

**Additional file  3.** Capture mark recapture with columns representing dates of capture and rows representing individuals.
